# Supplementary material for: Tuning magnetic spirals beyond room temperature with chemical disorder
Source: Nat Commun. 2016 Dec 16;7:13758. doi: 10.1038/ncomms13758 (PMC5171853; doi:10.1038/ncomms13758)
Supplement: Supplementary Information — Supplementary Figures, Supplementary Table and Supplementary References. [file ncomms13758-s1.pdf]

Supplementary Figures

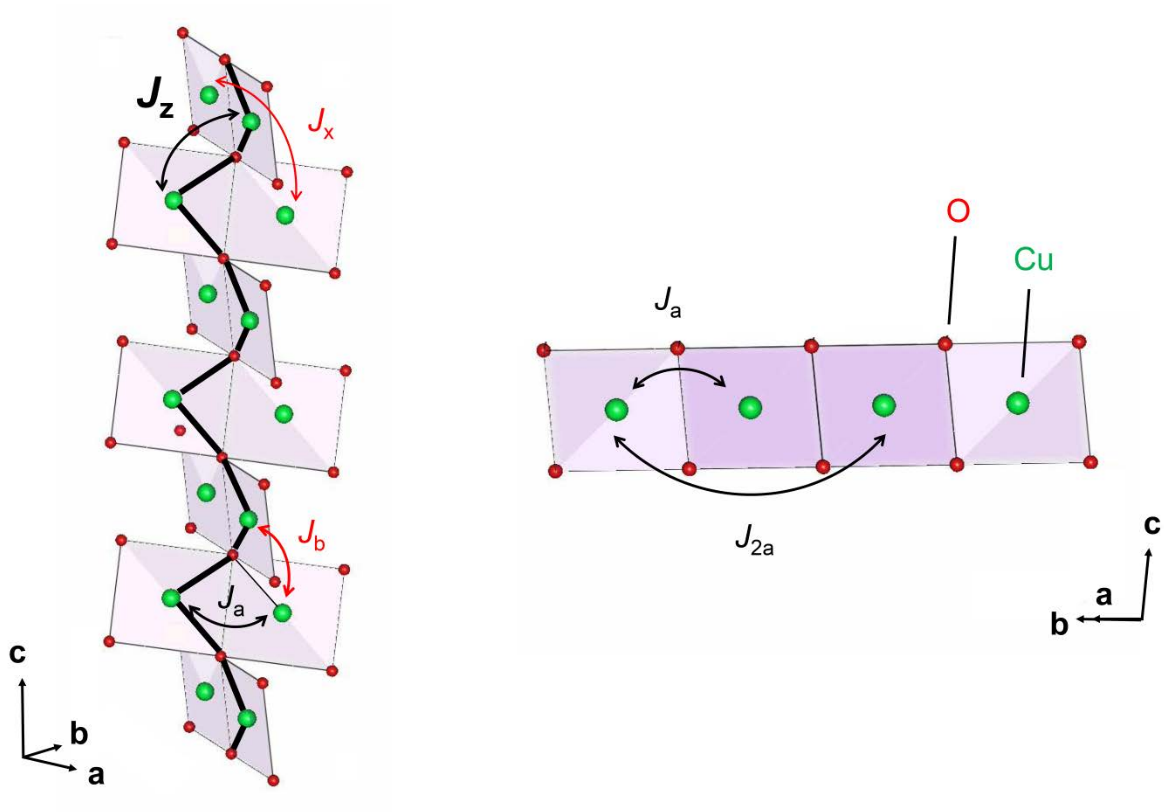

Supplementary Figure 1. Crystal structure of CuO. The figure illustrates the quasi-1D chain character of the exchange interactions. The NN superexchange couplings (J_z>0, J_a>0, J_x<0, J_b<0) together with the main super-superexchange coupling J_{2a}>0 are shown. As usual, negative couplings (in red) are FM and AFM couplings (in black) are positive. According with most authors[1-5], one of the 2 couplings along the chain axis (J_z, in bold) is at least ~ 4 - 5 times larger in absolute value that any other coupling in the structure.

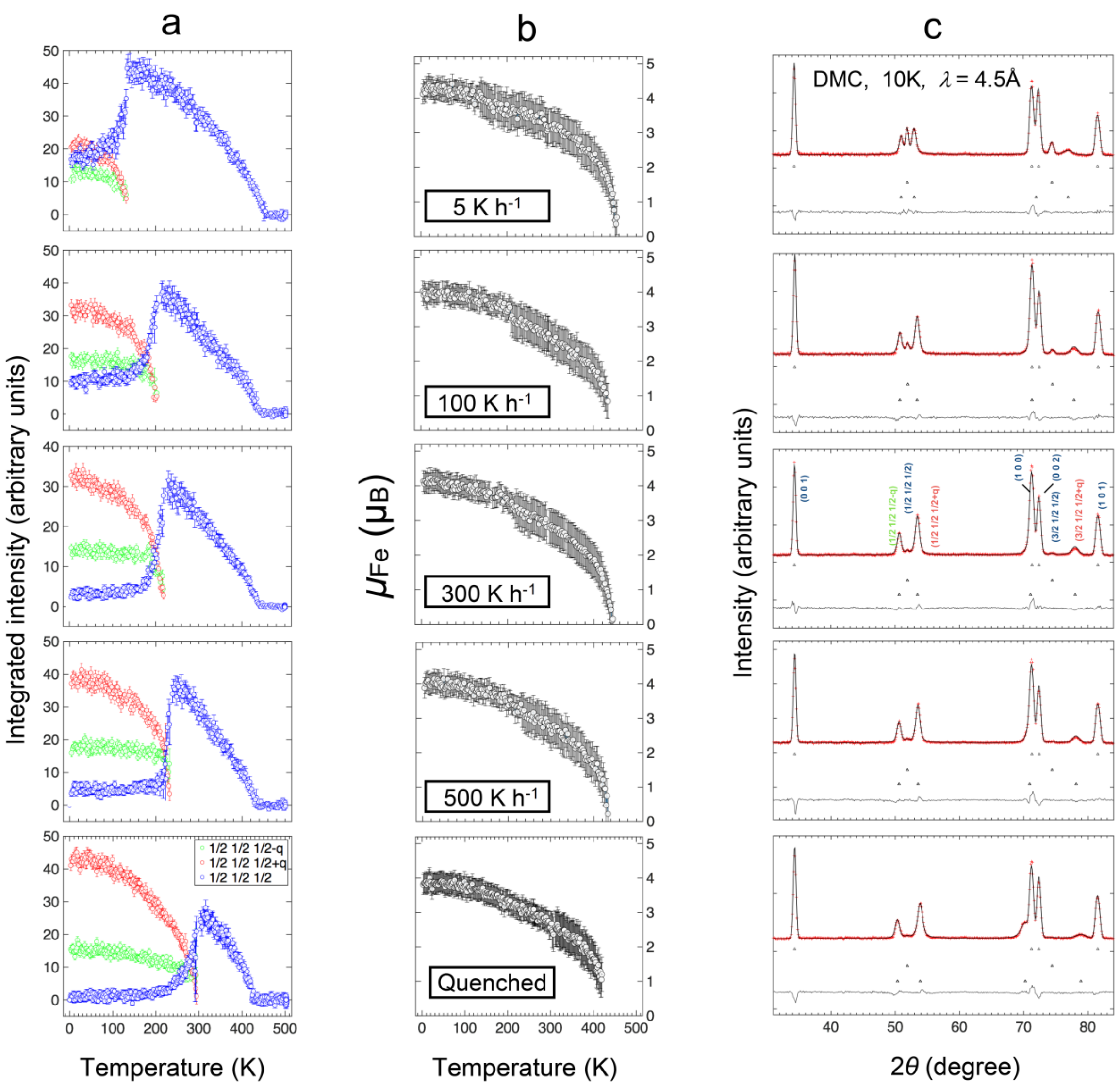

Supplementary Figure 2. Neutron diffraction analysis. a, temperature dependence of the commensurate magnetic reflection (½ ½ ½) and the income-mensurate satellites (½ ½ ½ ± *q*) for the five $YBaCuFeO_5$ samples synthesized with different cooling rates. **b,** temperature dependence of the Fe magnetic moment. **c,** Rietveld fits of the neutron powder diffraction patterns recorded for the 5 samples at 10K on the diffractometer DMC with λ = 4.5Å. The three rows of ticks (up to down) indicate respectively the positions of the Bragg reflections of the nuclear, collinear AFM and incommensurately modulated magnetic spiral phases. The error bars are the standard deviations obtained from 3-Gaussian least-square fits of the (½ ½ ½) Bragg reflection and its incommensurate satellites.

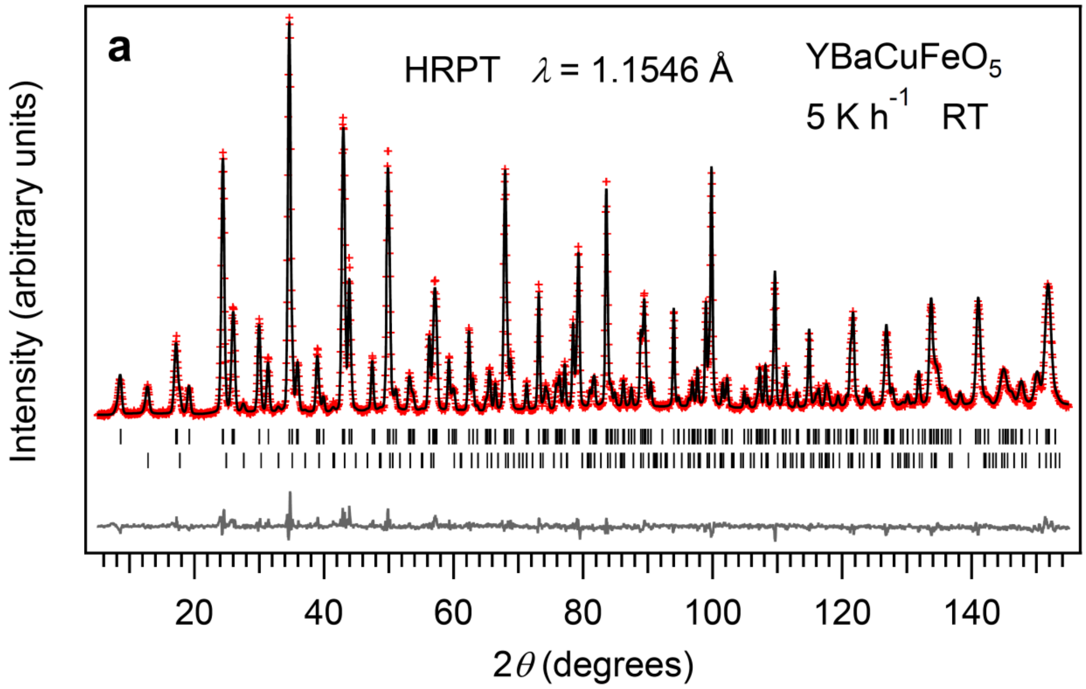

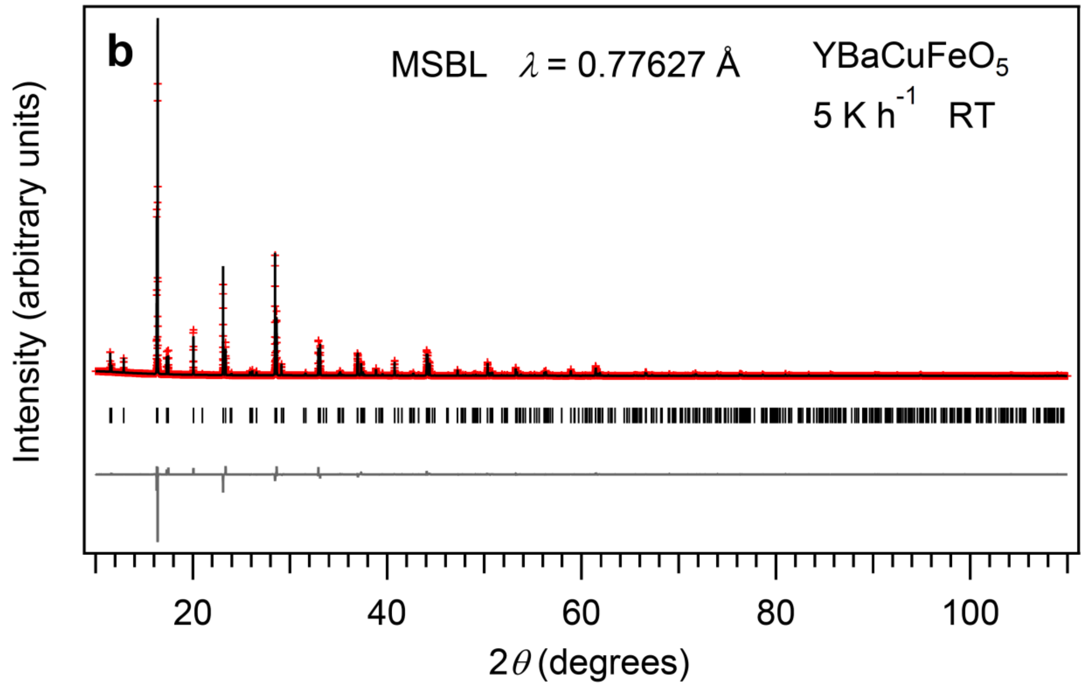

Supplementary Figure 3. Rietveld fits. The figure shows the neutron (**a**) and x-ray synchrotron (**b**) powder diffraction patterns of the $YBaCuFeO_5$ sample prepared with the slowest cooling rate (5 K h^{-1}). The data were collected at RT at the neutron diffractometer HRPT (SINQ), λ = 1.1546 Å and the Materials Science Beam Line (SLS), λ = 0.77627 Å. The second row of ticks in the HRPT pattern corresponds to the calculated positions of the magnetic Bragg reflections of the collinear magnetic structure.

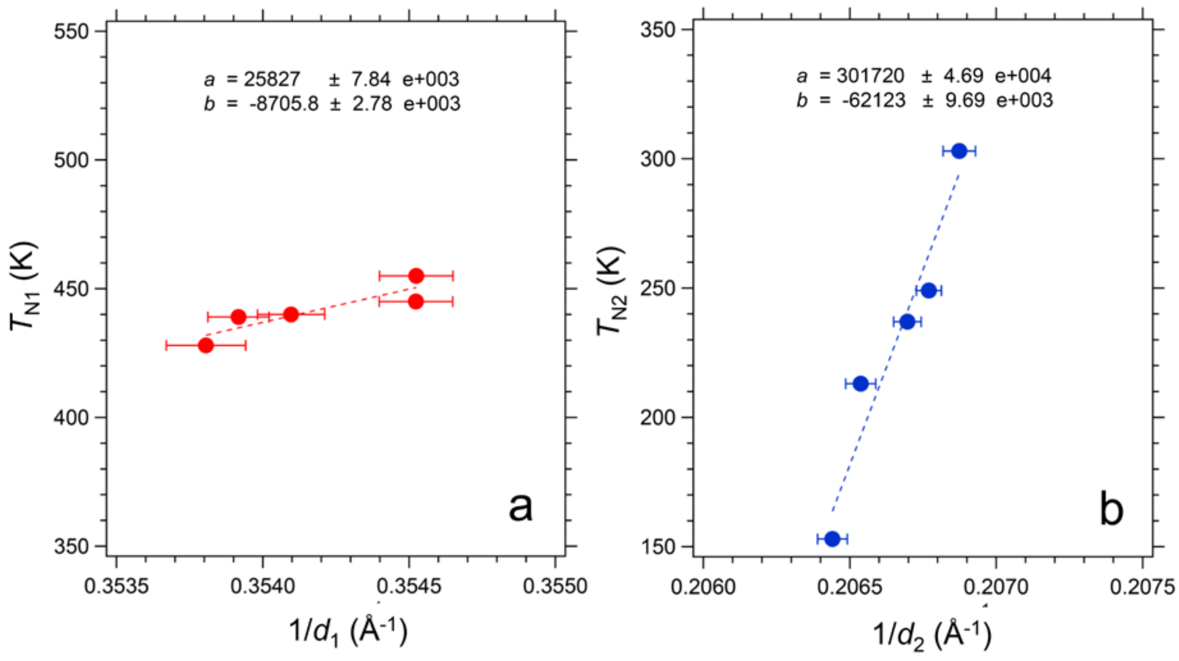

Supplementary Figure 4. Evolution of T_{N1} and T_{N2} with the inverse of the interatomic distances d_1 and d_2. Apart from a shift, the span of the left and bottom axes in the two figures are identical. The insets are the coefficients of the least-square linear fits shown in the figure ($T = a\ 1/d + b$). Note that in the case of T_{N2} the slope is about 12 times larger than for T_{N1}. The error bars on $1/d_1$ (**a**) and $1/d_2$ (**b**) were calculated applying standard error propagation formulas to the d_1 and d_2 standard deviations obtained from the Rietveld program FullProf[6,7].

Supplementary Tables

*P*4mm, 10K	5 K h^{-1}	100 K h^{-1}	300 K h^{-1}	500 K h^{-1}	Quenched
**a** (Å)	3.86905(1)	3.86923 (1)	3.86940 (1)	3.86951 (1)	3.86930 (1)
**c** (Å)	7.63792(4)	7.63546 (4)	7.63523 (4)	7.63500 (4)	7.63265 (4)
***Ba* 1*a* (0 0 *z*)**					
z	-0.004(2)	-0.003(3)	-0.005(3)	-0.001(3)	-0.004(2)
U_{11} (Å^2)	0.0021(3)	0.0026(2)	0.0022(3)	0.0029(3)	0.0021(3)
U_{33} (Å^2)	0.024(2)	0.0196(9)	0.024(2)	0.0192(10)	0.024(2)
***Y* 1a (0 0 *z*)**					
z	0.4948(12)	0.4946(13)	0.4934(12)	0.4964(11)	0.4948(12)
U_{11} (Å^2)	0.00226(19)	0.00264(17)	0.00259(17)	0.00293(19)	0.00226(19)
U_{33} (Å^2)	0.0039(4)	0.0029(4)	0.0027(4)	0.0043(7)	0.0039(4)
***Cu* 1*b* (½ ½ *z*)**					
Occ.	0.416(16)	0.424(21)	0.424(16)	0.464(16)	0.448(24)
z	0.2829(3)	0.2833(2)	0.2826(3)	0.2815(3)	0.2829(3)
U_{iso} (Å^2)	0.00152(11)	0.00149(10)	0.00185(10)	0.00224(11)	0.00152(11)
***Fe* 1*b* (½ ½ *z*)**					
Occ.	0.584(16)	0576(21)	0.576(16)	0.536(16)	0.552(24)
z	0.2544(2)	0.2543(2)	0.2547(2)	0.2557(3)	0.2544(2)
U_{iso} (Å^2)	0.00152(11)	0.00149(10)	0.00185(10)	0.00224(11)	0.00152(11)
**$O1_{apical}$ 1*b* (½ ½ 0)**					
z	0	0	0	0	0
U_{11} (Å^2)	0.0044(3)	0.0042(3)	0.0046(3)	0.0047(3)	0.0044(3)
U_{33} (Å^2)	0.0158(9)	0.0160(7)	0.0144(7)	0.0140(7)	0.0158(9)
**$O2_{basal}$ 2*c* (½ 0 *z*)**					
z	0.3113(8)	0.3107(11)	0.3105(9)	0.3108(13)	0.3113(8)
U_{11} (Å^2)	0.0031(2)	0.00333(19)	0.00371(20)	0.0040(2)	0.0031(2)
U_{22} (Å^2)	0.0027(2)	0.00252(18)	0.00281(19)	0.0039(2)	0.0027(2)
U_{33} (Å^2)	0.0067(3)	0.0071(3)	0.0072(3)	0.0053(3)	0.0067(3)
**$O2'_{basal}$ 2*c* (½ 0 *z*)**					
z	0.6803(8)	0.6795(11)	0.6794(9)	0.6796(13)	0.6803(8)
U_{11} (Å^2)	0.0031(2)	0.00333(19)	0.00371(20)	0.0040(2)	0.0031(2)
U_{22} (Å^2)	0.0027(2)	0.00252(18)	0.00281(19)	0.0039(2)	0.0027(2)
U_{33} (Å^2)	0.0067(3)	0.0071(3)	0.0072(3)	0.0053(3)	0.0067(3)
μFe / μCu (μ_B)	3.82(3) / 0.767(6)	4.02(6) / 0.81(1)	4.1(2) / 0.83(3)	3.9(2) / 0.80(3)	3.9(2) / 0.78 (4)
φ_G (degrees)	32(1)	38(2)	38(2)	43(2)	45(2)
q_G (recip. lattice units)	0.4203(4)	0.3943(3)	0.3888(3)	0.3840(3)	0.3603(4)
% spiral phase	76.9	90.9	97.9	98.3	98.9
**HRPT**					
Chi^2	2.24	2.25	2.49	2.28	2.13
R_p	3.65	3.48	3.76	3.49	3.62
R_{wp}	4.68	4.43	4.81	4.49	4.62
R_{Bragg}	2.62	2.41	2.22	2.63	2.98
**DMC**					
Chi^2	2.24	2.98	3.50	3.45	4.92
R_p	3.54	4.15	5.24	4.82	4.09
R_{wp}	4.49	5.22	6.61	6.36	5.38
R_{Mag} (spiral phase)	10.7	7.70	6.61	9.61	10.5

Supplementary Table 1. Crystallographic data. Results of the Rietveld fits of the HPRT and DMC neutron diffraction patterns of the five $YBaCuFeO_5$ samples at 10K.

Supplementary References

1. Shimizu, T., Matsumoto, T., Goto, A., Chandrasekhar Rao, T.V., Yoshimura, K. and Kosuge, K. Spin susceptibility and superexchange interaction in the antiferromagnet CuO. *Phys. Rev. B* **68**, 224433-224440 (2003).

2. Giovanetti, G., Kumar, S., Stroppa, A., van den Brink, J., Picozzi, S. and Lorenzana, J. High-T_C ferroelectricity emerging from magnetic degeneracy in cupric oxide. *Phys. Rev. Lett.* **106**, 026401-026405 (2011).

3. Forsyth, J.B., Brown, P.J. and Wanklyn B.M. Magnetism in cupric oxide. *J. Phys. C: Solid State Phys.* **21**, 2917-2929 (1988).

4. Hellsvik J., Balestieri M., Usui T., Stroppa A., Bergman A., Bergqvist L., Prabhakaran D., Eriksson O.,Picozzi S., Kimura T. and Lorenzana J. Tuning order-by-disorder multiferroicity in CuO by doping. *Phys. Rev. B* **90**, 014437-014445 (2014).

5. Rocquefelte, X., Schwarz K., Blaha P., Kumar S. and van den Brink J. Room-temperature spin-spiral multiferroicity in high-pressure cupric oxide. *Nature Communications* 4:2511 (2013).

6. Rodríguez-Carvajal J., Recent advances in magnetic structure determination by neutrón poder diffraction + FullProf, *Physica B* **192**, 55-56 (1993).

7. Rodríguez-Carvajal J., Recent developments of the program FullProf, *Commission of powder diffraction (IUCR), Newsletter No*. **26**, 12-19 (2001).
